# Home-administered transcranial direct current stimulation with asynchronous remote supervision in the treatment of depression: feasibility, tolerability, and clinical effectiveness

**DOI:** 10.3389/fpsyt.2023.1206805

**Published:** 2023-11-02

**Authors:** Theodoros Koutsomitros, Sandra A. Schwarz, Kenneth T. van der Zee, Teresa Schuhmann, Alexander T. Sack

**Affiliations:** ^1^Department of Cognitive Neuroscience, Faculty of Psychology and Neuroscience, Maastricht University, Maastricht, Netherlands; ^2^Greek rTMS Clinic, Medical Psychotherapeutic Centre (I.Ψ.K.), Thessaloniki, Greece; ^3^Institute of Psychotherapy, Medical Psychotherapeutic Centre (I.Ψ.K.), Thessaloniki, Greece; ^4^Donders Institute, Centre for Cognitive Neuroimaging, Radboud University, Nijmegen, Netherlands; ^5^Brain Imaging Centre (MBIC), Maastricht University, Maastricht, Netherlands; ^6^School for Mental Health and Neuroscience, Brain and Nerve Centre, Maastricht University Medical Centre, Maastricht, Netherlands

**Keywords:** transcranial electrical stimulation (TES), transcranial direct current stimulation (tDCS), home-administered, remotely supervised, asynchronous remote supervision, treatment resistant depression (TRD), major depressive disorder (MDD), depression

## Abstract

**Introduction (Background):**

Depression is an often chronic condition, characterized by wide-ranging physical, cognitive and psychosocial symptoms that can lead to disability, premature mortality or suicide. It affects 350 million people globally, yet up to 30% do not respond to traditional treatment, creating an urgent need for novel non-pharmacological treatments. This open-label naturalistic study assesses the practical feasibility, tolerability, and clinical effectiveness of home-administered transcranial direct current stimulation (tDCS) with asynchronous remote supervision, in the treatment of depression.

**Method:**

Over the course of 3 weeks, 40 patients with depression received psychotherapy and half of this group also received daily bi-frontal tDCS stimulation of the dorsolateral prefrontal cortex. These patients received tDCS for 30 min per session with the anode placed over F3 and the cathode over F4, at an intensity of 2 mA for 21 consecutive days. We measured patients' level of depression symptoms at four time points using the Beck Depression Inventory, before treatment and at 1-week intervals throughout the treatment period. We monitored practical feasibility such as daily protocol compliance and tolerability including side effects, with the PlatoScience cloud-based remote supervision platform.

**Results:**

Of the 20 patients in the tDCS group, 90% were able to comply with the protocol by not missing more than three of their assigned sessions, and none dropped out of the study. No serious adverse events were reported, with only 14 instances of mild to moderate side effects and two instances of scalp pain rated as severe, out of a total of 420 stimulation sessions. Patients in the tDCS group showed a significantly greater reduction in depression symptoms after 3 weeks of treatment, compared to the treatment as usual (TAU) group [t(57.2) = 2.268, p = 0.027]. The tDCS group also showed greater treatment response (50%) and depression remission rates (75%) compared to the TAU group (5 and 30%, respectively).

**Discussion (Conclusion):**

These findings provide a possible indication of the clinical effectiveness of home-administered tDCS for the treatment of depression, and its feasibility and tolerability in combination with asynchronous supervision.

## 1. Introduction

In 2019, one out of eight people globally lived with a mental health condition, including 280 million people diagnosed with major depressive disorder (MDD). Following the stress of the COVID-19 pandemic, depression diagnoses surged by 25%, reaching 350 million (1). Depression is characterized by a range of disabling physical, cognitive and psychosocial symptoms, such as changes in eating or sleeping patterns, concentration difficulties, loss of energy and motivation, and anxiety (2). Severe or recurrent depression often leads to serious social and vocational impairments, premature mortality due to its multiple comorbidities, or suicide (1). By 2030, the combined costs of mental health care, lost productivity and broader societal impacts are projected to cost the global economy up to US$6 trillion (1).

Current professional guidelines recommend a combination of psychotherapy and pharmacotherapy to treat depression (3), but only 42% of patients respond to first-line treatments (4). Up to 30% fail to improve after two courses of pharmacotherapy and are subsequently diagnosed with treatment-resistant depression (TRD) (5, 6). Complementary therapies such as meditation or lifestyle modifications have not been shown to be effective in TRD, so there is an urgent need for novel non-pharmacological treatments for those patients (7).

The underlying neurobiology of depression is not yet fully known, but atypical neural connectivity has been observed in patients (8). It is proposed that hypoactivity of the dorsolateral prefrontal cortex (DLPFC) diminishes the top-down ability to regulate emotions (9, 10), while hyperactivity of the limbic system increases negative bottom-up emotionality (11). In healthy individuals, the subgenual anterior cingulate cortex (sgACC) is negatively correlated with the DLPFC at rest and acts as a gatekeeper between the two networks (10). In depressed patients, however, this anticorrelation is exaggerated, and the sgACC amplifies bottom-up emotionality instead, which overwhelms the already hypoactive DLPFC (12, 13).

Non-invasive brain stimulation (NIBS) is an established, safe and effective non-pharmacological treatment for MDD (14) and TRD (15), that can be used on its own (16) or as an adjunct to psychotherapy (17, 18) or pharmacotherapy (19). It is hypothesized that NIBS treatment normalizes DLPFC function (10) by down regulating sgACC activity, which improves top-down emotional control in depression (12, 13, 20, 21). Neuromodulation can also induce lasting neuroplastic changes throughout interconnected brain networks (14, 16). The stimulation depolarizes neurons, which up-regulates long-term potentiation (LTP) and that leads to an enduring increase in signal transmission between simultaneously active neurons (22).

Transcranial direct current stimulation (tDCS) is a NIBS technique that generates a low intensity flow of direct electrical current in the neural tissue between two or more electrodes placed on the skull (23). In depression, anodal stimulation aimed at the left DLPFC is thought to improve top-down emotional control and connectivity (19, 22, 24, 25). A review of 27 randomized clinical trials (RCTs) with over 1,200 patients found significantly greater antidepressant effects with active- than sham-tDCS (26) and multiple studies have demonstrated that treatment effects can be maintained for at least six months (22, 27, 28). A consortium of experts recently analyzed these and other studies, rating the effectiveness of tDCS as “definitely effective” in the treatment of depression (Level A) (29).

In addition to its effectiveness, tDCS has been shown to be a safe and tolerable treatment for patients. A review of over 33,000 tDCS sessions with more than 1,000 participants, including children, older adults and patients with epilepsy, found a complete absence of serious adverse events (SAEs) (30). Other studies have reported only mild or transient discomfort, such headaches or skin sensations at the site of electrodes (31, 32). However, these have been observed to occur equally in the active- and sham-tDCS conditions (32) and participant attrition rates also tend not to differ significantly between active- and sham-tDCS groups (33).

The convergence of scientific evidence with the maturation of wearable tDCS headsets has created a convenient option for home-administered treatment, eliminating barriers such as cost, time or travel constraints during business hours. Previous research has shown that home-administered tDCS is a safe, tolerable and feasible treatment for patients with MDD (34) including also older adults (35). Other studies have shown significant antidepressant effects after six weeks of treatment with home-administered tDCS in TRD (36) and in MDD where the effects were also maintained for at least six months (37). Home-administered tDCS is already used as a treatment in a wide range of other neurological and psychiatric disorders, including schizophrenia, pain disorders, multiple sclerosis, dementia, Parkinson's Disease and stroke, with growing evidence of its safety, feasibility and effectiveness [see reviews (38–40)].

However, previous home-administered tDCS studies have reported limitations such as patients experiencing difficulties with manual electrode fixation and a lack of monitoring and guidance (34, 35, 41) as well as risks of over- or under-stimulation (42). These early studies demonstrate the advantages of standardization of montage and treatment by trained staff in research or clinical settings (34, 41). To address these limitations and improve the safety of home-administered tDCS, our study followed current TES safety recommendations including providing specifically tailored patient training, educational materials and support, in addition to ongoing compliance supervision and side effects monitoring (35, 41–43). The clinicians and researchers involved in our study attended an online “Transcranial Electrical Stimulation (TES) Practitioner Certification” training course, which focused on tDCS and was closely aligned with the International Federation of Clinical Neurophysiology guidelines (44).

Additionally, we used PlatoWork 2.0 tDCS headsets (PlatoScience ApS, Denmark) with a preconfigured fixed-montage of electrodes, which eliminates the need for manual measurement or fixation by patients (39). The headsets are a registered Class I medical device under the MDD 93/42/EEC standard and comply with current TES safety guidelines by including pre-programmed dose control of each stimulation session, an inbuilt impedance control that automatically terminates stimulation when electrode impedance exceeds 20 kΩ and three different options for the patient to safely discontinue stimulation (35, 41–43, 45).

Earlier home-administered tDCS studies used a range of supervision approaches such as home visits by researchers, sending photos of the montage, online symptom tracking and treatment diaries (34, 39, 40). In depression, recent tDCS studies used real-time video monitoring (37) or standardized participant training combined with session scheduling and regular assessments (35). The PlatoWork tDCS headsets are integrated with a cloud-based remote supervision platform, which collects data on protocol compliance, side effects and subjective ratings of sessions' feasibility and tolerability. Therefore, instead of real-time video monitoring synchronous with each stimulation session, our study used asynchronous daily monitoring of the remote supervision platform by a trained clinician. This approach allowed multiple patients to use their devices simultaneously, increasing flexibility for both the patient and the clinician, and reduced time demands for clinicians.

The aim of the current study was to test the feasibility and tolerability and clinical effectiveness of home-administered tDCS for the acute treatment of MDD. We conducted a three-week open-label naturalistic study with daily asynchronous supervision. We compared the effect of psychotherapy with the addition of home-administered tDCS, to treatment as usual (TAU) with psychotherapy only, on levels of depression. We hypothesized that all patients will show improvement in depression symptoms over time as a result of psychotherapy treatment, but that those receiving additional tDCS sessions will report a significantly greater improvement than the patients receiving TAU. We also hypothesized that home-administered tDCS treatment will be feasible and tolerable for patients, demonstrated by compliance with the treatment protocol and an absence of SAEs among reported side effects.

## 2. Materials and methods

### 2.1. Procedure

In this open-label naturalistic study, patients diagnosed with depression were recruited, screened for eligibility and asked for their written informed consent at the Medical Psychotherapeutic Centre in Thessaloniki, Greece, between November 2021 and October 2022. Subsequently, 40 patients received weekly, 45–60 min in-person psychotherapy sessions from a psychotherapist, clinical psychologist or psychiatrist, and completed the Beck Depression Inventory (BDI) four times, before treatment and then weekly for three weeks to measure their level of depressive symptoms.

Of these 40 patients, half were randomly assigned to receive home-administered tDCS treatment in addition to psychotherapy (“tDCS group”) and the remaining 20 continued with psychotherapy only as treatment as usual (“TAU group”). Stratified randomization was performed manually to create balanced groups controlling for the variables of age, sex, antidepressant medication use and the psychologist providing psychotherapy. Since this was an open-label naturalistic study in a clinical population, no sham-tDCS group was included.

Patients in the tDCS group were free to choose at what time of day they completed their daily tDCS session. All session data were automatically uploaded to the cloud-based remote supervision platform and asynchronously monitored each day by a trained clinician. Asynchronous supervision refers to remotely monitoring data within a 24-hour timeframe, compared to synchronous supervision such as real-time video monitoring, which is done simultaneously for the duration of the stimulation session. At the end of the study, patients returned the tDCS headsets to the clinic during their in-person psychotherapy session and were debriefed about the study.

### 2.2. Safety

The clinicians and researchers involved in our study attended the Certified TES Practitioner online training, which was closely aligned with the International Federation of Clinical Neurophysiology guidelines (44) covering the neurophysiology of tDCS, safety, hardware and software and clinical applications. Each patient in the tDCS group received an individual 20-minute in-person training session by a Certified TES Practitioner. Patient training included a demonstration of how to safely open, adjust, put on and remove the tDCS headset, how to use the integrated smartphone app to start, pause and stop the session, and also how to report side effects and submit feedback. Patients then received a digital copy of the 15-page PlatoWork instruction manual, which is also accessible within the app. Two patients requested additional support with downloading and setting up the app, and four were offered on-site support with their first treatment session. All remaining sessions were completed by patients at home.

At the end of each session, patients received a prompt in the app to report side effects and rate the stimulation session's tolerability and feasibility. Since this feedback was voluntary, there was an option to skip this prompt and patients did not always choose to submit a response (see Section 2.5). Reported side effects were tracked as per standard reporting guidelines (46). In the event that a SAE was reported, the clinician would contact the patient on the same day and the patient's access to the app would be remotely disabled, preventing further stimulation sessions until the clinician's approval. However, no SAEs were reported in this study.

### 2.3. Eligibility criteria

Patients were included in the study if they met the following inclusion criteria: (1) DSM-5 criteria for a depressive episode (296.22, 296.23 or 296.32, 296.33) as their primary diagnosis using the structured clinical interview for DSM-5 disorders—clinician version (SCID-5-CV); (2) ICD10 criteria for a moderate or severe depressive episode without psychotic symptoms (F32.1 or F32.3) or recurrent depressive disorder with a current moderate or severe depressive episode without psychotic symptoms (F33.1 or F33.2); (3) were aged between 20 and 55 years to ensure a comparable level of digital literacy; and (4) agreed not to modify their medications during the two weeks prior and the three weeks of tDCS treatment.

Patients were not included in the study if they met any of the following exclusion criteria: (1) primary diagnosis other than depression; (2) standard rTMS and tDCS contraindications (i.e., history of epilepsy, ferromagnetic head implants, history of neurosurgery, a pacemaker implant, patients with cranial or intracranial implants, patients without an intact skull, patients with skin conditions such as psoriasis); (3) use of medication known to substantially lower the seizure threshold (e.g., bupropion, clozapine); and (4) co-initiation of any new medication. Additionally, since previous studies (47, 48) showed that tDCS has lower antidepressant effects in patients who are taking concurrent benzodiazepine medication, (5) we also excluded any patient taking benzodiazepines.

### 2.4. Sample demographics

Forty patients were recruited by multiple practitioners within the same clinic and randomly assigned to one of the two groups, which had an equal allocation of 20 patients each and were closely matched in age. Although both groups appeared to differ in other characteristics, consistently with an open-label naturalistic study design, these differences were not statistically significant. All patients were aged between 20 and 55 years, with a slightly higher average age in the TAU group (36 years) than in the tDCS group (33 years), but this difference was not statistically significant. Of the total 40 participants, 27 identified as female, and thus the sex-ratio also differed between groups, but this difference was not statistically significant. Additionally, patients' depression type, their baseline depression level as measured by the BDI and the types of antidepressant medications used were also recorded, but no significant differences were found between groups, as shown in Table 1.

**Table 1:** Demographics of study sample.

**Measures**	**Mean/SD (range)**		**Group comparison**
	**TAU group**	**tDCS group**	
*N*	20	20	
Age	36.1/8.8 (20–55)	33.3/10.3 (20–55)	n.s. (*t* = −0.923, *p* = 0.362)
Sex	12 female/8 male	15 female/5 male	n.s. (*X*^2^ = 0.45584, *p* = 0.4996)
Depression type	13 single episode/7 treatment-resistant	12 single episode/8 treatment-resistant	n.s. (*X*^2^ = 0, *p* = 1)
Baseline depression level	22.9/7.8 (11–40)	22.0/8.8 (10–38)	n.s. (*t* = −0.377, *p* = 0.708)
Antidepressant use	5 none/4 SNRI/11 SSRI	5 none/4 SNRI/11 SSRI	n.s. (*X*^2^ = 0, *p* = 1)

NB. TAU, treatment as usual; tDCS, transcranial direct current stimulation.

Patients with bipolar depression were excluded from the study to minimize the risk of emergent manic or hypomanic episodes. Patients' comorbidities, in addition to a primary diagnosis of depression, are shown in Table 2. Particular care was taken to monitor all patients specifically for manic symptoms or increased suicidality, as per standard clinical care guidelines for any serotonergic antidepressant treatment, which applies also in unipolar depression (49).

**Table 2:** Comorbid disorders in study sample.

**Measures**	**Mean/SD (range)**		**Group comparison**
	**TAU group**	**tDCS group**	
Cyclothymia	20 no/0 yes	19 no/1 yes	n.s. (*X*^2^ = 0, *p* = 1)
Phobic anxiety disorder	19 no/1 yes	20 no/0 yes	n.s. (*X*^2^ = 0, *p* = 1)
Obsessive compulsive disorder	19 no/1 yes	20 no/0 yes	n.s. (*X*^2^ = 0, *p* = 1)
Anxiety disorder (unspecified)	18 no/2 yes	19 no/1 yes	n.s. (*X*^2^ = 0, *p* = 1)
Somatoform disorder	19 no/1 yes	19 no/1 yes	n.s. (*X*^2^ = 0, *p* = 1)
Personality disorder (specified)	14 no/6 yes	11 no/9 yes	n.s. (*X*^2^ = 0.42667, *p* = 0.5136)
Personality disorder (unspecified)	18 no/2 yes	15 no/5 yes	n.s. (*X*^2^ = 0.69264, *p* = 0.4053)

NB. TAU, treatment as usual; tDCS, transcranial direct current stimulation.

Of the 20 patients in the TAU group, five patients were not taking any antidepressants with the remaining 15 taking either serotonin-norepinephrine reuptake inhibitors (SNRIs; Duloxetine or Venlfaxaine) or selective serotonin reuptake inhibitors (SSRIs; Citalopram, Escitalopram, Fluoxetine, Fluvoxamine, Sertraline or Vortioxetine), as shown in Table 3. In the tDCS group, six out of the 20 patients were not taking any antidepressants, with the remaining 14 taking either SNRIs or SSRIs.

**Table 3:** Antidepressant medication usage in study sample.

**Medication**	**Treatment condition**		**Total**
	**TAU group**	**tDCS group**	
Citalopram	1	0	1
Duloxetine	2	1	3
Escitalopram	3	1	4
Fluoxetine	3	4	7
Fluvoxamine	1	1	2
Sertraline	1	2	3
Venlafaxine	2	3	5
Vortioxetine	2	2	4
None	5	6	11
Total	20	20	40

NB. TAU, treatment as usual; tDCS, transcranial direct current stimulation.

### 2.5. tDCS hardware and software

In this study, we used 20 PlatoWork 2.0 tDCS headsets (PlatoScience ApS, Denmark). The PlatoWork tDCS headset is a registered Class I medical device under the MDD 93/42/EEC standard, and complies with additional industry safety standards such as the Limited Output Transcranial Electrical Stimulation (LOTES) guidelines (45) and other TES safety recommendations (35, 41–43). The headset includes built-in safety features such as constant impedance control and current adjustment which automatically terminates the stimulation session if the electrode impedance exceeds 20 kΩ (41, 43) resulting in a prompt in the app for the patient to adjust the headset and start a new session. To ensure correct dose control, the protocol for session duration and stimulation intensity was pre-programmed remotely in accordance with the parameters described below (see Section 2.6) and patients did not have access to these settings in the app (42).

**Figure 1:**
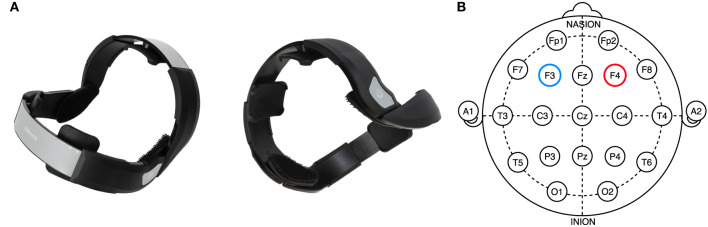
tDCS headset and electrode positioning. **(A)** Two figures depicting the PlatoWork 2.0 tDCS headset (PlatoScience ApS, Denmark). **(B)** A schematic showing the targeted stimulation locations of F3 and F4, with the anode in blue and the cathode in red, according to the International 10-20 EEG System. Images reproduced with permission from the manufacturer.

The PlatoWork tDCS headset is a fixed-montage clinical headset designed for home-administered use and does not require manual electrode measurement or fixation, as shown in Figure 1. The mechanical geometry of the headset mirrors the International 10-20 EEG System by following its angles and logic. This defines the fixed location of the three remotely-programmable electrodes that are integrated into the frame at the F3, F4 and Pz positions on the scalp. For our study, only two electrodes located at F3 and F4 were active, and the Pz electrode was disabled, as it is used only in alternative montages. The size of the headset scales to fit a variety of head sizes, with the relative positioning of electrodes remaining the same within and between patients, with the aim of providing consistent and reliable results across multiple sessions.

The headset is controlled by the patient through an integrated PlatoWork smartphone app, which includes a “Stimulate” button to start the pre-programmed session, a “Pause” button to stop the session temporarily and a “Stop” button that discontinues it permanently. The stimulation session is also automatically terminated when the headset is removed from the head. At the end of each session, the app prompted patients to report any side effects and submit a subjective rating of the session's feasibility and tolerability, on a five-point star-rating scale, where 1= Very Poor and 5 = Very Good. No personal patient data was collected by the device or stored in the cloud-based software. Only data related to the operation of the device such as session date, time and duration, and the current and impedance of each session, as well as patient feedback including side effects and ratings, was stored.

### 2.6. tDCS protocol

We used the recommended bi-frontal montage for the treatment of depression, with the anode at F3 aiming at the left DLPFC, and the cathode contralateral at F4 (24). The current was gradually ramped up and down for 15 seconds at the beginning and end of each session. Active stimulation was delivered at an intensity of 2 mA for 30 min once per day, for 21 consecutive days. Contrary to most other studies which deliver tDCS only on working days with a weekend washout period, the portable nature of the tDCS headset used here allowed for its use seven days a week.

Due to the low focality of tDCS technology (50) the International 10-20 EEG System was considered sufficient to use for targeting of the left DLPFC. This is also consistent with previous work in the field (47, 51–53). The consistency of targeting the same areas within and between participants was supported by the fixed montage headset since all three electrodes remained fixed in the same relative location for all sessions, even after scaling the headset to fit different head sizes.

### 2.7. BDI questionnaire

The Beck Depression Inventory (BDI) was used to assess patients' depression symptoms. The BDI is a 21-item multiple-choice self-report questionnaire that includes statements such as “I feel guilty all of the time” and “I don't get real satisfaction out of anything anymore”. Each item on the BDI is scored on a four-point scale from 0 to 3, where the maximum total score is 63, and scores of 29 or higher indicate a severe episode of depression (54) while remission is defined by scores below 13 (55). The BDI questionnaire was administered at the start of weekly psychotherapy sessions four times during the study with one week intervals.

### 2.8. Statistical analysis

A linear mixed model (LMM) was selected to investigate whether the level of depression differed significantly between the tDCS and the TAU group, and to analyze the nature of the interaction between treatment type over time and its effect on depression. The LMM was selected because it allows not only for the investigation of the fixed effects of each factor like a traditional analysis of variance (ANOVA), but to also vary the effect of a factor on a per-patient basis. Our model included the following factors: time, group (TAU or tDCS), age, sex, depression type (i.e., treatment resistance status), the antidepressant medication used by the patient (i.e., SSRI, SNRI or none) and the existence of comorbid disorders in addition to depression.

We used a backwards procedure to select the most parsimonious model that was not a significantly worse fit for the data, starting with the most complex model which included all the factors listed above as fixed factors with the addition of the interaction term to capture the hypothesized improvement in depression scores over time in both groups, as well as the random factor of patient to account for individual variation and a random slope of time assuming that each patient responded at a different rate. Using a Likelihood Ratio (LR) test, we selected the best fitting model. Since it included a significant interaction term, we conducted *post-hoc* Bonferroni-corrected pairwise comparisons to investigate at what time point the difference in depression levels between the two groups became significant.

The statistical analysis was conducted in R (56), using the lmer function for the LMM from the lme4 package (57), the anova function from core R to compare models (56) and the emmeans function from the emmeans package to conduct *post-hoc* pairwise comparisons (58).

## 3. Results

### 3.1. Practical feasibility and tolerability

Practical feasibility was defined as the extent of patients' compliance with the stimulation protocol. Of the 20 patients in the tDCS group, 90% demonstrated good compliance and did not miss more than three of their assigned 21 sessions, while half of the group completed all sessions with full 100% compliance. One patient confirmed having missed three sessions due to a COVID-19 infection. There was no attrition in the tDCS group but three (15%) of the TAU group patients dropped out due to their inability to comply with either the inclusion and/or exclusion criteria. They were replaced with three newly recruited participants to maintain two equal groups of 20 patients. Additionally, 100% of tDCS sessions were within the acceptable range of <20 kΩ impedance.

Practical feasibility and tolerability were also measured with patients' subjective rating of the session submitted in the app on a five-point scale, where 1 = Very Poor and 5 = Very Good. Of the total 420 assigned sessions, 91% of ratings from the 18 patients who chose to provide them, rated the sessions at four or five stars, with an average rating of 4.6 out of 5. None of the sessions received a rating of one star. The data of our study was also analyzed for usage of the Stop and Pause buttons and the removal of the headset to terminate a stimulation session early, but none of these options were used by any of the participants.

Tolerability was assessed using the side effects reported by patients and tracked according to standardized recommendations (46). Of the 20 patients in the tDCS group, 12 chose not to submit any feedback or report any side effects. The remaining eight patients (40%) submitted 30 instances of feedback of which 16 (53%) included side effects and the remaining 14 included generic positive feedback such as “Good” and “Okay”. Out of a total of 420 assigned sessions, the 16 instances of reported side effects, shown in Table 4, included 14 reports of mild to moderate discomfort such as “Slight headache, very slight, nothing extreme”. The most severe side effects were two instances of scalp pain. No SEAs were reported during the study.

**Table 4:** Reported tDCS side effects out of a total of 420 assigned sessions.

**Side effect**	**Not reported**	**Mild**	**Moderate**	**Severe**
Headache	418	2	0	0
Neck pain	420	0	0	0
Scalp pain	409	5	4	2
Tingling	419	1	0	0
Itching	420	0	0	0
Burning sensation	420	0	0	0
Skin redness	420	0	0	0
Sleepiness	420	0	0	0
Trouble concentrating	420	0	0	0
Acute mood change	420	0	0	0
Other: phosphene	419	1	0	0
Other: discomfort	419	1	0	0
Total:	–	10	4	2

### 3.2. Clinical effectiveness

Subsequent model iterations were compared, starting with the full model containing every fixed factor as well as the interaction between time and group, the random intercept of patient and the random slope of time. Table 5 shows the effects removed in each iteration and the statistical comparison to the model above it, using the log-likelihood ratio, the *X*^2^-test and its corresponding *p*-value. All effects, except for sex and the interaction term, do not significantly affect the model fit while making it more parsimonious, so they have been included in the final model in addition to the fixed effects of time and group. In summary, the final model contained the fixed effects of sex, time, group and the interaction between time and group, as well as the random intercept of patient.

**Table 5:** Linear mixed model iterations.

**Removed effect**	**log-likelihood**	***X* ^2^**	***p*-value**
Full model	−484.19		
Model without slope	−486.63	4.872	0.088
Model without any comorbidity	−486.97	0.682	0.409
Model without antidepressant type	−487.29	0.652	0.722
Model without depression variant	−488.56	2.528	0.112
Model without sex	−490.97	4.831	0.028
Model without age	−488.56	1.696	0.193
Model without interaction	−491.78	4.743	0.029

The fixed effects of time [*t*(118) = −5.904, *p* < 0.001], gender [*t*(37) = 2.173, *p* = 0.036] and the interaction between time and group [*t*(118) = 2.181, *p* = 0.031] on the level of depression, as measured by the BDI score, were all statistically significant. However, the fixed effect of group was not significant [*t*(72.044) = −0.032, *p* = 0.975], indicating that the effect of which treatment group patients were assigned to is significant only at specific time points during the study.

**Figure 2:**
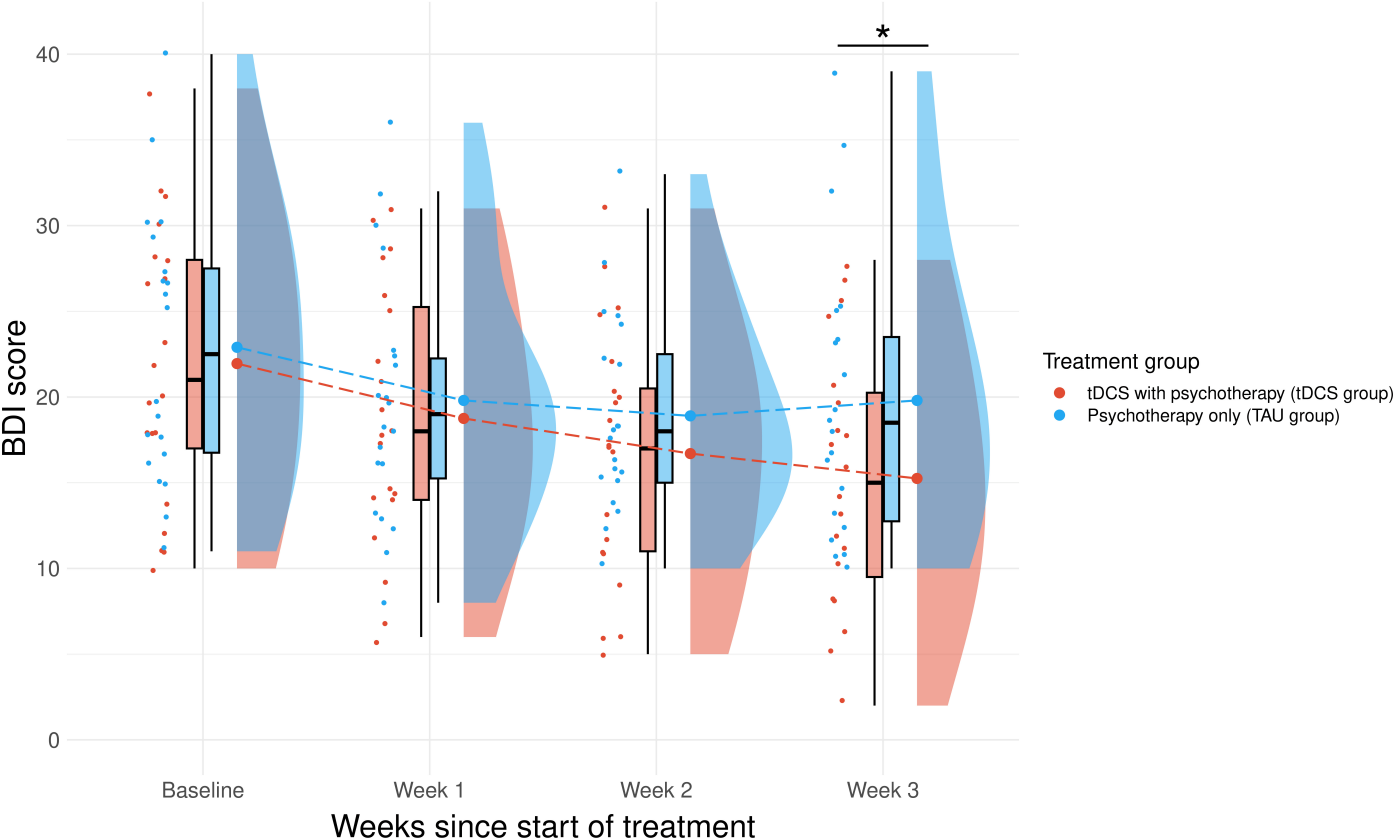
Change in BDI score comparing the score at baseline to the 3-week therapy. This interaction between the treatment group and the change in BDI score over time was significant, *t*(118) = 2.181, *p* = 0.031. Pairwise comparisons showed a significant decrease in BDI score when comparing the two groups at the 3 week timepoint [*t*(35) = 3.255, *p* = 0.038]. *Significant (*p* < 0.05) difference. NB. TAU, treatment as usual; tDCS, transcranial direct current stimulation.

To further investigate the nature of the significant interaction effect of time and group, *post-hoc* pairwise comparisons, corrected for multiple comparisons using the Bonferroni method, were conducted. As shown in Figure 2, we found that in the third week of the study, the tDCS group showed a significantly lower level of depression, as measured by the BDI score, than the TAU group [*t*(57.2) = 2.268, *p* = 0.027]. Comparisons of the two groups at baseline [*t*(57.2) = 0.719, *p* = 0.475], after one week [*t*(57.2) = 0.762, *p* = 0.449], and after two weeks [*t*(57.2) = 1.257, *p* = 0.214] did not generate statistically significant results, indicating that at least three weeks of treatment is required to produce clinically noticeable improvement in depression symptoms.

Finally, treatment response and depression remission rates were compared between the two groups. We defined treatment response as a decrease in BDI score of at least 50% from baseline, and remission as a BDI score below 13 (55). Our results show that the end of the study, only 5% of patients in the TAU group responded to treatment compared to 50% in the tDCS group. Similarly, the TAU group reached a remission rate of only 30% compared to 75% in the tDCS group.

## 4. Discussion

We conducted a three-week, open-label naturalistic study to investigate the feasibility, tolerability and clinical effectiveness of home-administered tDCS with asynchronous remote supervision as a treatment for depression. Our study compared the effect of tDCS in combination with psychotherapy, to TAU with psychotherapy alone, on the level of depression reported by patients. We expected that tDCS would be a feasible and tolerable treatment option for depression, and that patients in the tDCS group would report a significantly greater reduction in depression than those in the TAU group.

Extending on previous findings, our results suggest that home-administered tDCS with asynchronous supervision is a feasible and tolerable treatment (30–32). We defined practical feasibility as the level of compliance with the treatment protocol and found that out of the 20 patients in the tDCS group, 90% demonstrated good compliance by not missing more than three of their assigned 21 sessions, and no patients dropped out of this group. In comparison, the drop out rate of the TAU group was 15% due to three patients' inability to comply with either the inclusion and/or exclusion criteria. Additionally, 91% of ratings of the stimulation sessions' subjective feasibility and tolerability were either “Good” or “Very Good” with an average rating of 4.6 out of 5, with no record of any sessions being deliberately terminated earlier than scheduled. These results suggest that home-administered tDCS was easy to use and did not pose any significant practical challenges for the patients in our study.

Tolerability was also assessed by analysing self-reported side effects, which patients in the tDCS group submitted using their integrated smartphone app. Consistently with previous research on the safety of tDCS (30), no SEAs were reported during our study. There were 16 instances of reported side effects out of a total of 420 stimulation sessions assigned to the study. These included 14 instances of mild or moderate discomfort and only two instances of scalp pain that were rated as “severe”. Previous tDCS studies report similar side effect profiles, but these have also been observed to occur equally in the active- and sham-tDCS conditions (32). Our findings indicate that tDCS is a safe and tolerable treatment option for patients with depression.

In line with previous research, our pilot results also showed a significant reduction in depression symptoms after three weeks in the tDCS group compared to TAU, providing a possible indication of the clinical effectiveness of tDCS in depression (19, 22, 24, 25, 29) and particularly home-administered tDCS (34–37) with asynchronous supervision (35). Additionally, while in the TAU group only 5% of patients responded to treatment and 30% achieved remission from depression, patients in the tDCS group reached a much higher 50% response rate and a 75% remission rate. Altogether, our results allude to the possible benefits of home-administered tDCS with asynchronous supervision, including lower barriers to treatment, more flexibility for patients, fewer time demands for clinicians, and more robust research designs for researchers. However, caution is warranted when interpreting results from open-label naturalistic studies. Without a sham-tDCS condition, it is impossible to conclusively separate the treatment effects of the stimulation from patient expectations or other aspects of taking part in the study.

Our study encountered several limitations, which we hope can be rectified with further research. Firstly, open-label naturalistic studies such as ours are limited in the conclusions that can be drawn from their results. Our study lacked a sham-tDCS condition and did not control for psychotherapy type. We therefore recommend future studies use a sham-controlled design and either control for psychotherapy type or increase the sample size to limit its potentially confounding influence. Additionally, participants in the tDCS group were asked to voluntarily report side effects after each stimulation session, and many chose not to submit any feedback. We therefore recommend a systematic approach to capturing side effects before and after the session in a standardized questionnaire format (46).

Since previous studies have shown greater antidepressant effects with longer total tDCS stimulation duration (24, 25, 59), we also suggest a longer study duration to find the optimal treatment time for the maximum possible effect size, and a follow-up measure to investigate maintenance effects. Finally, we recommend the use of expert ratings such as the Hamilton Depression Rating Scale or the Montgomery-Åsberg Depression Rating Scale instead of or in addition to the self-reported BDI questionnaire, which we had to select due to COVID-19 restrictions on in-person gatherings. Looking into the future, tDCS could even be integrated with data from other wearables and self-report measures, to predict relapses of depression and remotely initiate maintenance treatment (28, 42, 60).

## 5. Conclusion

In conclusion, our study provides a possible indication of the clinical effectiveness of home-administered tDCS with daily asynchronous supervision, as a treatment for depression. Our results showed a clinically significant reduction in depression symptoms after three weeks of tDCS treatment with no serious side effects. The findings of our study also indicate that it is a feasible and tolerable treatment, that is accessible and flexible for patients and can be delivered by clinicians at scale to meet the surging global demand for depression treatment.

## Data availability statement

The raw data supporting the conclusions of this article will be made available by the authors, without undue reservation.

## Ethics statement

Ethical review and approval was not required for the study on human participants in accordance with the local legislation and institutional requirements. This was an open-label naturalistic study without a placebo group or experimental randomisation, and as such does not require ethical approval. This study was conducted in accordance with the local legislation and institutional data safety requirements. All participants in all conditions received their usual evidence-based treatment as prescribed by their clinician, and provided their written informed consent for their anonymised data to be used in scientific research.

## Author contributions

TK and AS: conceptualization, investigation, and resources. TK and TS: data curation. TK, KZ, and AS: formal analysis. TK, SS, KZ, TS, and AS: methodology, writing—original draft, and writing—review & editing. TK and SS: project administration and project management. TS and AS: supervision. TK: validation. TK and KZ: visualization. All authors have read and agreed to the published version of the manuscript.
